# Mechanisms of HIV Protein Degradation into Epitopes: Implications for Vaccine Design

**DOI:** 10.3390/v6083271

**Published:** 2014-08-21

**Authors:** Marijana Rucevic, Julie Boucau, Jens Dinter, Georgio Kourjian, Sylvie Le Gall

**Affiliations:** Ragon Institute of MGH, MIT and Harvard, Massachusetts General Hospital and Harvard Medical School, Cambridge, MA 02139, USA; E-Mails: Rucevic.Marijana@mgh.harvard.edu (M.R.); jnoucau@mgh.harvard.edu (J.B.); jdinter@mgh.harvard.edu (J.D.); gkourjian@mgh.harvard.edu (G.K.)

**Keywords:** HIV, antigen processing, protein degradation, proteasome, aminopeptidase, peptidase, immunogen, vaccine vector, dendritic cells, T cells, viral evolution

## Abstract

The degradation of HIV-derived proteins into epitopes displayed by MHC-I or MHC-II are the first events leading to the priming of HIV-specific immune responses and to the recognition of infected cells. Despite a wealth of information about peptidases involved in protein degradation, our knowledge of epitope presentation during HIV infection remains limited. Here we review current data on HIV protein degradation linking epitope production and immunodominance, viral evolution and impaired epitope presentation. We propose that an in-depth understanding of HIV antigen processing and presentation in relevant primary cells could be exploited to identify signatures leading to efficient or inefficient epitope presentation in HIV proteomes, and to improve the design of immunogens eliciting immune responses efficiently recognizing all infected cells.

## 1. Antigen Processing Pathways

Protein degradation by the antigen processing machinery is a continuous process regulating all cellular functions by defining the lifespan of all proteins, discarding misfolded proteins, recycling amino acids to make new proteins, and initiating immune monitoring [1].

Proteins and misfolded or incomplete translation products such as defective ribosomal products [2] are degraded by proteasomes, the most ubiquitous cellular proteases. Proteasomes are made of 4 rings, 2 outer rings of 7 distinct alpha subunits and 2 inner rings of 7 distinct beta subunits, including 3 catalytic subunits cleaving after hydrophobic, acidic or basic residues respectively, allowing for the cleavage of all substrates [3,4]. The 20S core proteasomes can be fitted with regulatory lids leading to the assembly of 26S proteasomes. The 19S lid interacts with E3 ubiquitin ligases and polyubiquitinated substrates and contributes to unfolding substrates before degradation and opening of the proteasome chamber. Whereas 26S proteasomes degrade mostly polyubiquitinated substrates, 20S proteasomes and proteasomes capped with regulatory PA28/11S lids are able to degrade non-polyubiquitinated substrates. Besides constitutive proteasomes expressed in all cell types, immunoproteasomes are present in lymphoid tissues and can be induced in other cell types upon infection or cytokine exposure [5]. Interferon gamma induces the expression of catalytic immunosubunits and PA28 lids leading to the assembly of 20S and 26S immunoproteasomes [6]. All proteasomes degrade proteins into fragments ranging from a few residues up to 32 aa long, including epitopes, peptides containing epitopes (epitope precursors) and also amino acids to be recycled for protein synthesis [7]. Although peptides generated by various types of proteasomes overlap, they may also create unique peptides [7,8]. Immunoproteasomes tend to produce longer peptides ending with C-terminal hydrophobic residues, the most frequent C-terminal anchor for most MHC-I isotypes, and may favor the production of epitopes [9]. The presence of both proteasomes and immunoproteasomes in the same cell is associated with a greater variety of peptides available for presentation [10]. Degradation products from the proteasome can be further degraded by proteasomes as well as other cytosolic peptidases including endopeptidases TPPII [11,12], thimet oligopeptidases (TOP) [13,14,15], leucine aminopeptidase (LAP) [11,12], puromycin sensitive aminopeptidase (PSA) [16], bleomycin sensitive hydrolase (BH) [17], insulin degrading enzyme [18] or nardilysin [19]. Each peptidase has specificities in terms of substrate length and residues but they are not always well defined. The contribution of post-proteasomal peptidases is variable according to epitopes [18,19], sometimes controversial [20,21,22], and the order in which they cleave substrates is rarely defined [23,24]. Cytosolic peptidases cannot unfold proteins and therefore degrade post-proteasomal degradation products. Post-proteasomal peptidases such as TOP can produce epitopes from longer precursors or destroy peptides, thus contributing to defining sequences and the amount of peptides available for presentation to T cells [15,25]. Cytosolic peptides of mostly 8–16 aa long with adequate anchor residues for the transporter associated to antigen processing (TAP) [26] are translocated into the endoplasmic reticulum (ER) where peptides can be trimmed by ER-resident aminopeptidases ERAP1 [21,27,28,29] and ERAP2 [30] before loading onto MHC-I. Exogenous antigens such as proteins, dead cells, antibodies-coated viruses can be endocytosed or phagocytosed and degraded by various cathepsins [31,32], proteases and IRAP [33] in endosomes and lysosomes. Some degradation peptides can be transferred back in the cytosol or ER for further trimming and cross-presentation by MHC-I. In cells expressing MHC-II a specific lysosomal compartment called MIIC enables loading of peptides onto MHC-II molecules after removal of the invariant chain [34]. Although MHC-II epitope processing is mostly accomplished by endolysosomal proteases, proteasomes may be involved in the production of MHC-II epitopes of endogenous origin. The distinction between endogenous and exogenous processing pathways is becoming more tenuous as many combinations of peptidases and compartments may be involved in the presentation or cross-presentation of peptides [35]. Additionally, the variable intracellular stability of peptides prior to loading onto MHC-I alters the amount of peptides available for MHC-I presentation [36,37]. Altogether the location, trafficking path and the presence or absence of cleavable motifs by various peptidases will shape the degradation patterns of proteins [38].

## 2. Degradation of HIV Proteins into Epitopes

Despite rather extensive knowledge of protein degradation pathways and high-throughput methods to sequence HIV in large cohorts of patients, our capacity to rapidly identify or predict T cell epitopes in pathogen-derived proteins after infection or vaccination is still cumbersome, mostly relying on screening of T cell responses by Elispot using PBMCs from infected persons. The identification of potential anchor residues for various MHC-I isotypes in HIV sequences shows that there are more putative epitopes than actual immune responses, suggesting limitations in either production of peptides, their presentation by MHC or available TCR repertoire. We still do not know which HIV peptides are actually processed and presented by HIV-infected cells. These studies are still technically difficult due to the high number of infected cells required for peptide extraction. The first evidence of presentation of HIV peptides by HIV-infected cell lines came through acid elution and HPLC purification of peptide fractions reactive against HIV-specific CD8 T cells [39]. A second study using HIV-infected cells secreting soluble HLA (to enhance the amount of HLA collected and sensitivity of the assay) identified by mass spectrometry numerous self-derived peptides eluted off soluble HLA molecules, and showed changes in the amount or identity of self-derived peptides. It did not lead to the identification of HIV peptides, possibly due to HIV peptide presentation being below the threshold of detection by mass spectrometers [40]. Another study identified HIV-derived MHC-II peptides presented by DC endocytosing p24 coupled to anti-DEC205 antibody [41]. Increased sensitivity of new mass spectrometers and various improvements in assays and mass spectrometry data analysis software will probably lead to the identification of a landscape of HIV and self-derived epitopes presented by HIV-infected cells in a near future. However, MHC-bound peptides from HIV-infected cells will always be limited by the combination of HLA of the donor and the technological limit of detection of peptides. It is necessary to better understand how HIV proteins are degraded into peptides in cells to evaluate the capacity of proteins (or immunogens) to generate peptides compatible with loading onto MHC-I or MHC-II, and the impact of cell type or infection on HIV epitope presentation. In addition to epitopes encoded by the conventional reading frames of HIV genes several groups identified cryptic epitopes derived from the degradation of HIV translation products derived from alternate reading frames of HIV shifted by 1, 2 or 3 bases during translation [42]. Cryptic epitopes elicited CTL responses and immune pressure leading to HLA-restricted intraepitopic mutations [43,44], including some impairing processing [45]. Whether these immune responses contribute to reducing viral load in natural infection or should be included in vaccine immunogens is still unknown. This new category of HIV-derived epitopes highlights our limited knowledge of the source of HIV epitopes and peptides presented by HIV-infected cells.

To study epitope production and presentation several experimental systems have been developed. The use of HIV-infected cells as targets for assays measuring HIV-specific T cell functions (killing, proliferation, cytokine production) measures both endogenous processing and presentation of epitopes and variability of MHC-peptide-TCR, and is limited by the availability of HIV-specific T cell clones. *In vitro* degradation of HIV proteins by purified proteasomes [46,47,48], ERAP1 [49,50] or by specific purified cathepsins [51] enables detailed studies of degradation patterns and cleavage preferences by specific peptidases. *In vitro* proteasomal degradation of Nef showed production of numerous peptides in epitope-rich areas of Nef enriched in hydrophobic residues [46]. Degradation of HIV p24 or p17 by proteasomes and ERAP1 also led to the efficient production of peptides containing immunodominant epitopes [49,50]. Degradation of HIV Env by purified cathepsins showed efficient production of epitopes (compared to proteasomal degradation) that may contribute to elicitation of CD4 T cell responses [51]. Single peptidase degradation facilitates the assessment of mutations on the production of epitope precursors or epitopes at a specific step of antigen processing [50,52]. However since protein degradation is a continuum involving multiple proteases (or several variants of proteasome species such as 20S/26S in the same cell), this approach may not permit the investigation of the complete processing of an epitope. Additionally, peptidases are often purified from cell lines that may not always be the most relevant to HIV infection. The use of intracellular compartments for protein degradation (cytosol, endosomes, lysosomes) allows us to account for all peptidases present in a given compartment and a given cell type or infection (although we cannot completely rule out that the preparation of subcellular fractions by damaging the normal structure of compartments may have some impact on protein degradation kinetics or patterns). The degradation of HIV proteins or long peptides in cytosolic extracts of primary cells showed that the kinetics of epitopes is variable even among overlapping epitopes [53,54], and that the efficiency of production of epitopes (timing and amount) is defined by motifs located within and outside epitopes [47,49,53,55,56], and the intracellular stability of peptides before loading onto MHC [36].

HIV virions enter cells by fusion at the plasma membrane and by endocytosis or antibody-mediated phagocytosis for immune complexes. Vaccines such as viral vectors or nanoparticles decorated with peptides or proteins enter dendritic cells by endocytosis. Degradation of HIV Envelope after deglycolysation showed poor proteasomal degradation and efficient degradation by cathepsins, in accordance with intrinsic differences in antigen degradation according to peptidases’ substrate preferences [51]. Side-by-side degradations of HIV proteins in cytosolic and endolysosomal extracts from primary cells showed that some MHC-I epitopes are similarly processed in the two pathways while others are better processed in one of them [57]. These results suggest that the mode of entry of HIV virions or immunogens, in addition to the subset of target cells, will affect production and presentation of HIV epitopes as shown in non-HIV models [58,59].

A corollary to studies on efficiency of epitope production is to better understand mechanisms leading to impaired epitope presentation in the context of viral evolution. HLA-restricted mutations occur frequently in HIV-infected individuals during acute and chronic infection [60,61,62,63,64,65]. Some of these mutations are induced by immune pressure to avoid epitope presentation or correspond to mutations restoring viral fitness [66,67,68,69,70,71,72,73]. Many HLA-restricted mutations within epitopes impair binding to MHC-I or to the TCR of CD8 T cells [67,74,75]. Mutations outside epitopes may affect the trimming of extended peptides into epitopes or change the degradation of long peptides by proteasomes [45,76,77,78,79,80]. Two recent studies identified motifs corresponding to antigen processing mutations at the population level by defining residues that can or cannot be cleaved by families of peptidases or group of peptidases in a subcellular compartment. One study identified residues that can be variably cleaved or not cleaved by aminopeptidases (some of which had been previously identified [81,82]), and demonstrated that the presence of poorly or non cleavable motifs introduced near an HIV epitope reduced or abolished epitope production *in vitro* and presentation to T cells [55]. In a population of over 1000 HIV-infected persons, N-flanking mutations evolved mostly toward poorly cleavable residues, showing that antigen processing mutations are frequent and can be predicted [55]. In another study that defined motifs linked to intracellular peptide stability or instability, a number of intraepitopic HLA-restricted mutations identified in a large population of HIV-infected persons (21 out of 25 mutants tested so far) led to decreased cytosolic stability and decreased the amount of peptide presented to CTL [36]. Thus, HIV has developed many ways to block epitope presentation through peptide destruction or through impaired trimming of long peptides into peptides better suited for MHC-I binding [83], leading to suboptimal presentation of peptides to CD8 T cells and impaired clearance of infected cells.

The accumulation of data on protein degradation by purified proteasomes led to the development of prediction tools such as NetChOP or PaProC to define potential cleavage sites in proteins of interest [84,85,86,87]. Although they do not account for the impact of infection or specifics of proteasomes in relevant primary cells they are valuable tools to identify potential proteasomal cleavage sites in an antigen. Improvements to these predictors for epitope processing came by incorporating binding of proteasomal degradation peptides to TAP for translocation into the ER, and more recently by combining proteasomal degradation, TAP binding and ERAP1 trimming to identify putative epitopes or the potential impact of HLA-restricted mutations on epitope processing [50,52,88]. A prediction tool for cytosolic peptide stability [36], which incorporates degradation by various PBMC cytosolic peptidases, could be included as an additional factor influencing the amount of peptides available for presentation. Our even more limited knowledge of MHC-II HIV epitope production and the loose and promiscuous binding of peptides to MHC-II renders the prediction of MHC-II epitopes more difficult. Considering the importance of CD4 T helper responses to elicit sustainable CD8 T cell responses, and the intriguing elicitation of MHC-II-restricted CD8 T cells responses after vaccination with an attenuated CMV vector expressing SIV proteins [89], predictors for MHC-II epitope processing will be necessary; so far only a few predictors of peptide binding to a few MHC-II alleles are available [90,91,92]. The current datasets available on protein degradation are still insufficient to build a complete predictor recapitulating protein degradation and epitope presentation by MHC-I or MHC-II alleles.

## 3. Antigen Processing in the Context of HIV Infection

HIV infection induces massive changes of cellular metabolism [93] and creates unique conditions for antigen processing and presentation in term of targeted cell subsets and conditions for protein degradation. Whether HIV epitope production in HIV infection or during preventive vaccination of healthy donors, or therapeutic vaccination of HIV-infected persons leads to the production of similar epitopes and priming of protective immune responses is not known.

HIV infects CD4 T cells, monocytes, macrophages and dendritic cells but we do not know if they similarly process and present HIV epitopes during infection. Whereas all cells contain proteasomes and post-proteasomal peptidases, their levels of expression and hydrolytic activities in primary cell subsets from a given individual are poorly defined. We showed that primary CD4 T cells present lower proteasomal and aminopeptidase activities than monocytes isolated from the same donor [54]. These differences affected the rate of degradation of long HIV peptides into epitopes, the kinetics of epitope production and the amount of epitopes and extended epitopes produced. *In vitro* degradation of HIV p24 by purified proteasomes from mature DC or activated CD4 T cells showed differences in the degradation patterns of p24 and production of HIV epitopes, supporting intrinsic differences in the hydrolytic activities of peptidases from different cell subsets [48]. HIV can enter cells by fusion at the plasma membrane or by endocytosis. The proportion of the two entry modes in various cell types is not well defined, not exclusive and may affect the degradation of incoming virions in infected cells. However, we are still lacking direct identification and comparison of HIV peptides presented by all HIV-infectable cell subsets and how potential differences may affect the timing and recognition of infected target cells by HIV-specific T cells.

HIV infection profoundly affects the transcriptional programs of cells [93] and triggers a cytokine storm including production of interferon alpha, gamma and interleukins. Interferon gamma induces the expression of catalytic immunosubunits of proteasomes toward immunoproteasomes and increases the expression of several aminopeptidases (LAP, ERAP1), TAP and MHC molecules [94,95]. TNF or LPS can also induce the formation of immunoproteasomes, but the impact of other cytokines or combination of cytokines and chemokines on the antigen processing machinery is not well defined [96]. Cytokines, virus or TLR ligands such as LPS or HIV RNA binding TLR7/8 trigger maturation of dendritic cells and macrophages. Maturation of dendritic cells reduces their antigen processing activities and modifies epitope presentation as shown previously indirectly through epitope-specific T cell activation assays in various infection models [97,98,99,100,101].

HIV Protease inhibitors (PI) are designed to block HIV protease catalytic sites to prevent HIV maturation into fully infectious particles. First generation PIs such as Saquinavir and Ritonavir present some cross-reactivity with proteasome catalytic sites and alter proteasome activity *in vivo* in mice as well as in human cells *in vitro* and LCMV-specific T cell responses in treated mice [102,103,104,105,106]. A recent study showed that HIV PIs affected not only proteasome but also aminopeptidase activities in various ways according to drug and concentration in human PBMC. HIV PIs modified HIV peptide degradation and epitope production, both in *in vitro* degradation assays in extracts treated with PIs, and in HIV-infected cells endogenously processing and presenting HIV peptides to HIV-specific T cells [107].

Besides these external stimuli, HIV infection itself affects antigen processing. In cells isolated from HIV-infected donors CD4 T cells present lower activities than monocytes as seen in cells isolated from healthy donors. Peptidase activities in cells from HIV-infected donors were heterogeneous with a trend toward decrease in CD4 T cells and increase in monocytes compared to healthy donors [54]. The very low number of circulating HIV-infected cells and variable cellular activation levels in patients may contribute to the heterogeneity of antigen processing activities measured in patients’ PBMC. *In vitro* HIV infection alters antigen processing activities in monocyte-derived macrophages [108,109]. Interestingly, the entry mode of HIV (infection or receptor-mediated endocytosis of naked virus or AT-2 inactivated virus, complement-mediated opsonization particles) variably affects peptidase activities and subsequently epitope presentation to T cells, at least in *in vitro* studies [109,110,111]. *In vitro* studies revealed that uptake of HIV Tat altered epitope processing activities and viral epitope presentation but the impact on the processing of HIV is not known [112]. However, uptake of HIV proteins, antiretrovirals or cytokine-induced modifications may alter antigen processing in neighboring uninfected cells in HIV-infected persons.

## 4. Exploiting Assays and Knowledge of HIV Antigen Processing and Processing for Improved Vaccine Design

A commonly accepted approach to HIV vaccine design (including prophylactic vaccines protecting from infection or therapeutic vaccines clearing or controlling established infection) would combine a potent antibody response to prevent establishment of infection and sustainable T cell responses to control viral replication [113]. Challenges faced by vaccinologists include the genetic diversity of HIV, our lack of understanding of protective immunity (since there is no case of natural clearance of HIV infection), the establishment of viral reservoirs that cannot be eliminated with antiretroviral therapy, and our inability to induce B cells making specific neutralizing antibodies [114]. Current areas of focus in vaccine development examined in *in vitro* studies, preclinical animal studies and clinical trials include designing and testing various types of immunogens (proteins, peptides, nucleic acids) [115], viral vectors (non-replicative or attenuated persistent) [116,117], vectorless system (nanoparticles carrying immunogen) [118], and adjuvants [119]. Since current clinical trials and vaccines eliciting antibodies are addressed elsewhere in this journal issue this review will focus on important notions to consider in the design of vaccines eliciting T cell immunity.

HIV infection leads to a predictable hierarchy of T cell responses with narrow immunodominance of one or several T cell responses for each HLA type [64,120]. Immunodominance of T cell responses is defined as the most frequent T cell immune responses (or T cell responses eliciting the highest production of interferon gamma) in a population sharing a given HLA [121,122]. Since HIV is not cleared by immune responses elicited during infection, a vaccine leading to the same immunodominance patterns as natural HIV infection will probably not be successful at preventing or clearing HIV infection. Two indirect lines of evidence support the need of breaking natural immunodominance during vaccination. First the Merck STEP clinical trial based on an adenovirus 5 vector expressing full HIV proteins elicited T cell immune responses in vaccinees similar to those elicited during natural HIV infection. However these responses failed to protect vaccinees from HIV infection or to reduce viral load in those who became infected [123]. Secondly an attenuated Rhesus CMV vector expressing complete SIV proteins (*i.e.*, live persistent virus with attenuated capacity to replicate) led to clearance of high or low dose SIV infection in 50% of Rhesus macaques up to 3 years after infection. The attenuated vector led to broad CD4 and CD8 T cell responses with an effector phenotype that were detected both in blood and lymph nodes of vaccinated monkeys [124,125,126]. Intriguingly, most SIV-specific CD8 T cell responses elicited by the vaccine recognized MHC-II-restricted epitopes [89]. The mechanisms leading to elicitation of unconventional immune responses in all vaccinated Monkeys, their potential role in the clearance of infection in 50% of monkeys are still unknown. Finally the presence of dominant immune responses with limited antiviral capacity and the association between subdominant immune responses and lower viral load in HIV-infected persons [127] support the hypothesis that breaking natural immunodominance may enhance the efficacy of HIV vaccines [128]. As both attenuated CMV vectors and adenoviral vectors expressed complete SIV or HIV proteins, breaking immunodominance could be accomplished through the use of specific viral vectors. However a better understanding of the effect of vectors on epitope presentation and priming is required. Factors shaping immunodominance in various viral infections include—alone or in combination—kinetics of processing and presentation of epitopes, affinity for HLA or the TCR and the TCR repertoire [49,53,129,130,131,132]. The identification and fine mapping of HIV-specific naïve and memory CD4 T cell helper responses in unexposed donors showed that 83% of epitopes share homologies with microbial organisms (which may have primed these preexisting HIV-specific responses). These intriguing results suggest that the immune repertoire prior to HIV infection may contribute to shaping immunodominance of HIV immune responses during infection [133].

Breaking immunodominance could be accomplished through different approaches. Eliminating known immunodominant epitopes through deletions or mutations preventing presentation [134], or limiting the immunogen to portions of HIV proteins assembled in a chimeric protein [135] broke natural immunodominance and led to different hierarchies of immune responses after vaccination. These immunogens did not prevent establishment of infection but offered partial protection after challenge with a Vaccinia virus-derived vector expressing HIV in a mouse model [134]. Another approach to modify immunogens, successfully used to shift immunodominance in other viral infections in mice is to introduce mutations in region flanking epitopes so as to increase the production of some epitopes and decrease the production of others [136,137,138,139,140,141]. The introduction of cleavable or poorly cleavable motifs around HIV epitopes increased or decreased epitope processing by 10- to 100-fold and could be used to optimize epitope presentation [47,53]. In the absence of better understanding of protective immune responses the benefit of these approaches is still limited but they provide ways to test and modify epitope production and presentation and to refine the design of vaccine immunogen before *in vivo* validation.

The high variability of HIV in the infected population and across clades may require either the design of vaccines specific for clades or to focus the immune responses on the most conserved areas of the virus [115]. The emergence of escape mutations in acute HIV infection is largely predictable for many HLA which can be exploited in vaccine strategies. If vaccines elicit immune responses not only against consensus HIV sequences but also against frequent HLA-restricted mutants this will create additional pressure on the virus that will be forced to evolve toward variants with low replicative capacity. Mosaic immunogens made of a combination of distinct immunogens containing either HIV consensus sequences of Gag, Pol, Env (mosaic 1) or sequences carrying increasing number of frequent mutations (mosaic 2 or 3) have been computationally engineered and inserted into adenoviral vectors [142]. These mosaic vectors used in prime boost combinations elicited SIV-specific CD4 and CD8 T cell and antibody responses, and significantly reduced the per-exposure risk of acquisition of SIV [143]. Whether mutations introduced in mosaic 2 may alter immunogen degradation and lead to the presentation of new epitopes is not known but may broaden immune responses after vaccination. The opposite and complementary approach to the mosaic is the design of immunogens limited to the most conserved areas of several HIV proteins and when needed the most frequent variant of the sequence so as to cover >98% of HIV sequences [135,144,145]. Conserved sequences constitute vulnerable areas of the virus that can be identified in a linear fashion by sequence comparison in cohorts of HIV-infected persons [146], or as sectors of HIV proteins that coevolve at multiple sites under a given HLA pressure [73,147]. Peptides corresponding to conserved areas can be assembled in a chimeric protein by addition of linkers that do not create new epitopes based on the presence of potential anchors but introduce efficient cleavage sites for liberation of HIV fragments. These immunogens used in DNA prime/boost vaccination elicited CD4, CD8 T cell and antibodies responses in mice and Macaques and will eventually be tested in clinical trials [148,149]. Alternatively, peptides from conserved areas of HIV proteins may be used in peptide-based vaccines where peptides and adjuvants are electroporated [150] or carried to lymph nodes by nanoparticles [151]. In addition to conserved and variable areas of HIV proteins, cryptic epitopes generated from alternate reading frames could potentially be included in HIV immunogens, provided that immune responses against these epitopes are shown to contribute to reducing viral load.

The choice of vectors and adjuvants and the specific subset of dendritic cells endocytosing the vaccine and could also alter conditions in which the peptides are processed [119,152,153,154]. Adjuvants such as CpG, TLR ligands will trigger changes in hydrolytic activities of endolysosomal peptidases, epitope production and presentation, and possibly affect priming of immune responses [155,156,157]. Virus-derived vectors such as adenovirus or CMV could elicit or recall T cell immunity against these viruses and may alter the overall hierarchy of immune responses against the immunogen after vaccination. Replication-competent viruses used as persistent vectors might interact with the antigen processing machinery and possibly affect production of epitopes. The elicitation of MHC-II-restricted CD8 T cell responses by attenuated CMV vectors expressing SIV antigens in vaccinated monkeys sheds light on our limited understanding of conditions established by vectors for priming of immune responses but open new avenues to manipulate immunodominance and immunity during vaccination [158].

Therapeutic vaccines aiming at clearing or substantially reducing HIV infection is urgently needed to replace lifelong treatment of the HIV-infected population. One approach consists in combining reactivation of HIV provirus with drugs such as HDAC inhibitors with vaccines boosting HIV immune responses in HIV-infected persons receiving ART [159,160]. Latent infection of CD4 T cells, provirus reactivation and ART might create unique conditions for the processing and presentation of HIV epitopes. Defining epitopes presented in the context of latency (if any) and of reactivation will be necessary to better define epitopes and corresponding immune responses that should be boosted by therapeutic vaccines.

Further studies are needed to identify protective immune responses for prophylactic and therapeutic vaccines, specifically with regards to epitope specificity covering various HLA, ratio or phenotypes of CD4 and CD8 T cell responses, and how to specifically induce them during vaccination. Guiding principles such as encompassing viral diversity or focusing on conserved areas, adequate matching epitope presentation between infected cells and DC receiving vaccines are defined, and multiple tools to study epitope production and immune responses are available. Regardless of the vector or adjuvants used for vaccination, the HIV immunogen will have to be processed in professional antigen presenting cells for the priming of immune responses. It is necessary to ensure that—in the context established by the specific vaccination strategies discussed above—this HIV immunogen will be properly degraded into MHC-I and MHC-II epitopes corresponding to sustainable protective immune responses able to recognize infected cells. Assays to follow protein degradation into epitopes and motifs to optimize epitope provide a way to test epitope production from vaccine immunogens before larger studies in animal models and clinical trials, and guidelines to the design of immunogens leading to optimized epitope presentation.
